# In Situ Use of Mining Substrates for Wetland Construction: Results of a Pilot Experiment

**DOI:** 10.3390/plants13081161

**Published:** 2024-04-22

**Authors:** Carmen Hernández-Pérez, Salvadora Martínez-López, María José Martínez-Sánchez, Lucia Belén Martínez-Martínez, María Luz García-Lorenzo, Carmen Perez Sirvent

**Affiliations:** 1 Department of Agricultural Chemistry, Geology and Pedology, Faculty of Chemistry, University of Murcia, 30100 Murcia, Spain; carmen.hdz.perez@gmail.com (C.H.-P.); salvadora.martinez@um.es (S.M.-L.); mjose@um.es (M.J.M.-S.); lbmm@um.es (L.B.M.-M.); 2 Department of Mineralogy and Petrology, Faculty of Geology, Complutense University of Madrid, 28040 Madrid, Spain; mglorenzo@geo.ucm.es

**Keywords:** wetland, potentially toxic elements, acid mine drainage, coastal areas, mining sites, phytoremediation, metallophytes

## Abstract

This paper evaluates an experimental wetland as part of a pilot soil reclamation project in a mining area. The wetland was constructed using materials of mining origin from the area; most reactive materials of acid pH were stabilised using limestone filler. The study selected macrophytes that are tolerant to potentially toxic elements (PTEs) and resistant to salinity, namely *Phragmites australis*, *Juncus effusus*, and *Iris pseudacorus*. These macrophytes were then placed in pots containing substrates composed of different mixtures of topsoil, peat, and mining waste (black or yellow sand). A thorough analysis of the physicochemical and mineralogical characteristics of the materials included studies of PTE mobilisation. This study emphasises the significance of the rhizosphere in directing the transfer of PTEs to the plant and the correlation between the substrate and the development of plant defence mechanisms, such as the formation of Fe-plates. Scanning electron microscopy was used to highlight these aspects and validate the results of the analytical determinations. These wetlands can be proposed as a phytoremediation strategy for areas affected by mining and maritime influence. They are easy to construct and remain stable, providing important ecosystem services such as the natural attenuation of acid mine drainage, support for vegetation development and fauna, and a clean ecosystem.

## 1. Introduction

Mining activities can significantly impact the Earth’s surface, including the soil, water, and air [1,2,3,4], and it is crucial to implement appropriate measures to mitigate these detrimental consequences. If not, adverse effects such as soil degradation [5] and changes in biodiversity [6,7] will be observed. Remediation, repair, recovery, rehabilitation, and restoration, collectively known as the R4 strategy, are measures that can counteract these negative effects [8]. However, differentiating between the nuances of these four terms when referring to the elimination of mining effects can be challenging. When dealing with large areas, such as natural zones affected by mining, it is important to consider multiple strategies to minimise impacts and reduce risks. Such strategies may involve more than one approach to make the impacts acceptable. Objectivity should be maintained, and subjective evaluations should be avoided.

Pyrite oxidation in mine wastes can lead to acid mine drainage (AMD), which can significantly impact ecosystems. This may involve supergene alteration, acidification, increased cationic mobility, and the formation of soluble phases with varying hydration levels, known as efflorescences [9]. In semi-arid regions, AMD is linked to water availability and only becomes apparent during rainfall events, which are common in semi-arid Mediterranean climates. During mining activities, runoff water can carry particulate matter and soluble material, including potentially toxic elements (PTEs) [10,11]. It should be noted that the pH values of these waters can range from 2 to 6.

Coastal wetlands are particularly vulnerable to this problem because particulate inputs from mining and AMD can cause high concentrations of bioavailable PTEs in the sediment receiving zone [12]. The vegetation in these areas is predominantly halophilic, but in some cases, however, there is no vegetation, and the area is characterised by sediments with an acidic pH, a high PTE content, and fine granulometry. The mineralogical composition is similar to that of mining waste exposed to supergene alteration. One effective form of natural attenuation is runoff water from nearby carbonate soils, which releases calcium carbonate to neutralise AMD and precipitate PTEs [13,14]. This process occurs spontaneously during ecosystem recovery. However, even in these cases, high As contents and concentrations of organic matter can pose a potential threat to human health and the ecosystem [15]. Environmental conditions cause the reduction of As (V) to As (III), which increases its toxicity to the environment [16].

A successful long-term solution for mine sites affected by acid drainage is the construction of wetlands. Wetlands are flooded lands with water depths typically of less than 0.6 m. They contain emergent plants and water flows continuously, and the free surface remains close to ground level. These systems are complex, as they involve the exchange of nutrients between soil and biota in dilute media and sometimes in a reducing environment. Most surface soil horizons are typically under aerobic conditions [17,18,19], which is not the case of the wetland used here. This difference significantly affects the transport and uptake of nutrients through the plant root system, leading to both physiological and morphological adaptations.

Artificial wetlands are widely used for passive mine water treatment due to their advantages over other options. However, they are not a universal solution and may not be suitable in some cases [20,21]. Therefore, special care should be taken in the design of the wetland, the selection of building materials, the type of AMD to be treated, and the flow rate of the effluent. Passive technologies for treating AMD include aerobic wetlands, anaerobic wetlands (compost), and anoxic limestone drains. In aerobic wetlands, metal oxides and hydroxides undergo oxidation and precipitation reactions. Anaerobic wetlands promote bacterial activity that reduces sulphates, leading to the precipitation of metal sulphides and alkalinity generation. Anoxic limestone drains generate alkalinity and can be used as an AMD pretreatment.

The selection of water treatment technology depends on the type of water that requires treatment. For AMD, the most frequently used technology is a combination of an active permeable barrier and an aerobic wetland to immobilise large amounts of trivalent iron [22]. Caution should be exercised when recommending anaerobic conditions, as safe levels for certain elements, such as arsenic, may not always be achieved. Wetlands are complex ecosystems that involve several processes, including filtration, adsorption, plant uptake, and biogeochemical precipitation. It is important to control the parameters that define these structures [17,21,23,24,25,26,27,28,29,30] to ensure their proper functioning. Plant species play a specific role in the immobilisation of metals, their genotype, and environmental conditions determining the effectiveness of their action. Some plants are very tolerant to high metal concentrations but show negative effects on growth [17,31,32]. Removal rates of heavy metals in AMD by wetlands can be as high as 100%, although the rate is sometimes slow [11].

The objective of this study is to assess an experimental wetland that forms part of a soil remediation project at a coastal mining site. The study compares different substrates made from materials from the affected area. Additionally, the study investigates the selection of plant species that can thrive in areas with high PTE concentrations and can accumulate and tolerate heavy metals such as Pb, Zn, Cu, and Cd in their tissues. The wetland ought to be constructed on stabilised mining soils. It should trap dissolved and particulate metals and act as a barrier to prevent AMD-bearing runoff from reaching the sea. The funding of this case study could be applied to the final parts of wadis, which contribute water and particles with a high PTE content and acid pH during rainfall events, contaminating already remediated areas.

### Background

The Sierra Minera of Cartagena-La Unión, located in southeastern Spain, has two slopes that descend towards the sea. One slope is steep and has short, torrential streams that flow into small coves or into the bay of Portmán. The other slope has a longer course that flows into the Mar Menor (a marine coastal lagoon). Both slopes are characterised by the transportation of mining materials. For many years, numerous watercourses served as channels for the disposal of waste resulting from the washing and concentration of metallic minerals. Their beds are formed in many places by sediments similar to those found in mine tailings ponds located near mineral installations. During rainfall events, acidic water flows into the watercourses, and if the flow is not very high, natural attenuation occurs, neutralising the pH and precipitating Fe(III) in the form of hydroxide, the calcium carbonate present being the responsible agent.

Various publications have discussed the recovery options for Portman Bay, including studies and experiments related to the remediation of runoff waters that mix with acid mine drains and reach the beach area [10,15,33]. The meteoric waters are contained within the exposed section of the bay, resulting in the formation of a shallow lagoon during wet periods. The lagoon is home to various species, such as *Phragmites australis*, *Juncus effusus*, *Suaeda maritima*, *Arthrocnemum macrostachyum*, and *Tamarix boveana*, which are typical of hypersaline environments. To ensure the natural evolution of a highly degraded ecosystem, the intervention measures described herein involved constructing an aerobic wetland on soils treated with limestone filler.

## 2. Results

### 2.1. Characterisation and Selection of the Substrate Used

Materials from Portman Bay were used in the construction of the experimental wetland, acting similarly to the technosoils used in the Bay reclamation pilot project [34]. The more reactive and acidic pH material, known as yellow sand (YS), was previously amended with limestone filler [35]. The other material used was a more stable and a coarser textured material known as black sand (BS) and was used without amendment. The pots for this experiment were filled according to the instructions in the Section 3, using black sand (BS), yellow sand (YS) stabilised with limestone filler (LF), topsoil (TS), and peat.

The limestone filler (LF) selected as a pH stabilising agent had the following mineralogical composition: calcite (87%), illite (10%), and amorphous (3%). Table 1 summarises the physical and chemical characteristics of the filler used, its chemical composition, and PTE content. Its high pH, low EC, and low PTE content (concentrations below the limit of quantification) gave it suitable properties for use as a stabiliser in this experiment. Another important feature for the filler selection was the geographical proximity which, in this case, was also favourable (less than 30 km from the experimental area). The sustainability of an environmental project is a priority factor when selecting materials.

To select the topsoil, samples were taken from various cultivated soils near the experimental area with similar macromorphological characteristics and low organic carbon content. A soil with low salt content, basic pH, and silty-sandy texture was chosen. The mineralogy comprised calcite (10–15%), illite (40–50%), kaolinite (12–15%), clinochlore (8–10%), quartz (15–20%), and hematite (1–2%). The soil presented geogenic values in potentially toxic elements (PTEs) that exceed the reference values proposed for soils with similar mineralogy in this area [36] (Table 2, Table 3 and Table 4). This suggests that the soil is influenced by the Sierra Minera [37].

The black sand used in the experiment had a basic pH value, which indicates that the heavy metals were in precipitated phases. Therefore, they were not soluble or available through transfer pathways. The PTE content was high, with small variations from one sample to another. The granulometry of the sample was characterised by a coarse, sandy texture, with a low proportion of clay and silt-sized particles. The organic matter content was below the limit of quantification, as shown in Table 2.

The mineralogy of the black sand indicates that it has undergone a grain selection process, resulting in the presence of resistant and stable minerals such as quartz, iron minerals (siderite and goethite), and phyllosilicates (muscovite, chlorite, and greenalite). Pyrite and magnetite were also present, albeit in low proportions and with a regular distribution. Apart from siderite, which may be a neoformation mineral [11], the remaining identified phases were minerals inherited from the materials extracted from the mine. The mineralogy of black sands contained a higher proportion of inherited phases compared with previous materials, such as phyllosilicates, magnetite, pyrite, and hematite. These minerals were predominantly coarse in size (see Table 3).

Yellow sand was a reactive and poorly drained material that promoted the formation of soluble hydrated sulphates, known as efflorescence. These sulphates, which include Fe^+3^ and Al^+3^, as well as Fe^+2^, Mg^+2^, and Zn^+2^, along with varying amounts of water molecules, appear during the dry season of the year. The most frequently identified phases were copiapite, coquimbite, and bianchite [33,38]. These efflorescences can retain varying amounts of potentially toxic elements in their structure and arise due to capillarity and upward washing when there is no drainage. They crystallise on the surface, forming crusts and clusters of great variety and spectacularity, with colours ranging from white to orange, sometimes with greenish and ochre tones. The mineralogical composition and physical-chemical characteristics of the mineral flotation sludges are related to the process of supergene alteration. These sludges were dumped into the emerged zone of the bay without direct contact with seawater and without grain selection. As a result, the materials had an acidic pH and high conductivity, and they possessed a fine texture, silty clay, and high plasticity. The minerals muscovite, quartz, magnetite, and pyrite were inherited and belong to the gangue that accompanied the sulphides. Goethite and hematite may be of mixed origin, either alteration or inherited. Pyrite was responsible for the formation of jarosite and an acidic pH.

### 2.2. Study of Potential and Natural Mobilisation in the Base Materials (BS and YS)

**Figure 1:**
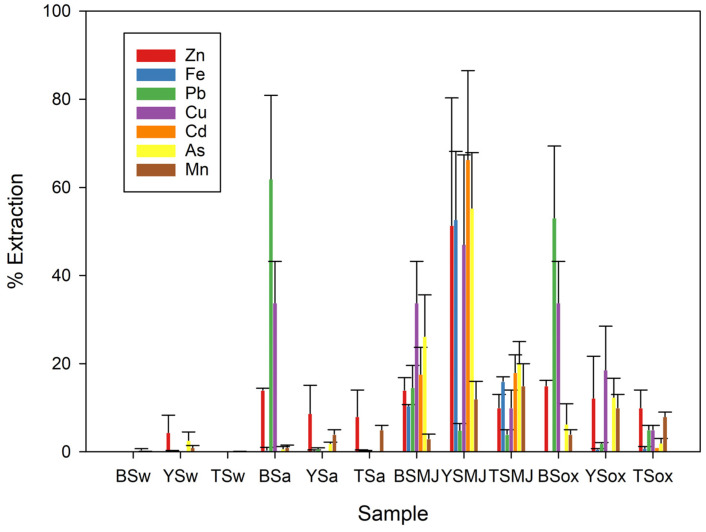
Average PTE extraction rates in selected soils (black sand (BS), yellow sand (YS), and topsoil (TS)) for media simulating different environmental conditions (w = water medium, a = acidic medium, MJ = complexing and reducing medium, ox = oxidising conditions).

Figure 1 summarises the results of the PTE mobilisation study in various media that simulated different environmental conditions for the topsoil, black sand, and yellow sand. The results allow for the estimation of the materials’ behaviour under different environmental conditions, predicting future changes. The study distinguishes between natural mobility conditions, such as rainwater (w) and acidic medium extractions (a), and potential conditions, such as complexing and reducing conditions, Mehra–Jackson extraction (MJ), and oxidant condition (ox).

At neutral pH, the mobility of potentially toxic elements (PTEs) in water was below the limit of quantification (QL) for black sands (BSw). In yellow sands (YSw), low mobility was observed for Zn and As, and it was below QL for the remaining PTEs. These conditions reflect the equilibrium established between the substrate and the aquatic environment and correspond to natural mobility.

Under acidic conditions with a pH below 2.5, mobility increased for both coarse and fine materials. However, coarse materials exhibited higher mobility than fine materials. The elements with the highest mobility were Pb, Cd, and Zn. This behaviour can be attributed to the presence of siderite and other carbonates that solubilise under these pH conditions. Under acidic conditions, which simulate high AMD inputs in the absence of limestone materials that could buffer the acidity, BSa showed greater mobilisation than YSa, resulting in significant amounts of soluble Pb, Cu, and, to a lesser extent, Fe.

In complexing and reducing media, using the Mehra–Jackson method to simulate anaerobic conditions or high organic matter (OM) content, both materials showed significant mobility, the yellow sands being the most affected. In these conditions, the elements that exhibited the highest mobility were Zn, Cu, As, Cd, and Fe. The phases of Fe(III) oxyhydroxides present were reduced by releasing retained metals into the crystalline network. As exhibited higher mobilisation rates than Fe in both materials (BSMJ and YSMJ), and so these conditions should be excluded when managing the use of these materials to prevent arsenic contamination.

Finally, under oxidising conditions that simulate supergene alteration, the black sands were found to be more sensitive than the yellow sands. The acidic medium, which is characteristic of these conditions, dissolved siderite and solubilised Pb, Cu, Fe, and As. In the BSox samples, Cu, Fe, As, and Mn were mobilised but to a lesser extent than in the YSox samples.

Table 3 shows that no phases of Pb, As, or Cd were identified in the samples studied. Therefore, these metals are included in the crystalline network of identified minerals and are insoluble under neutral pH conditions and oxygenated media. All of this means that the reactivity of the particles is much greater in the finer particle sizes found in landfills. However, there is a very low mobilisation of these particles as they are immobilised by carbonates and iron oxides and hydroxides.

### 2.3. Results of the Monitoring Plan

**Figure 2:**
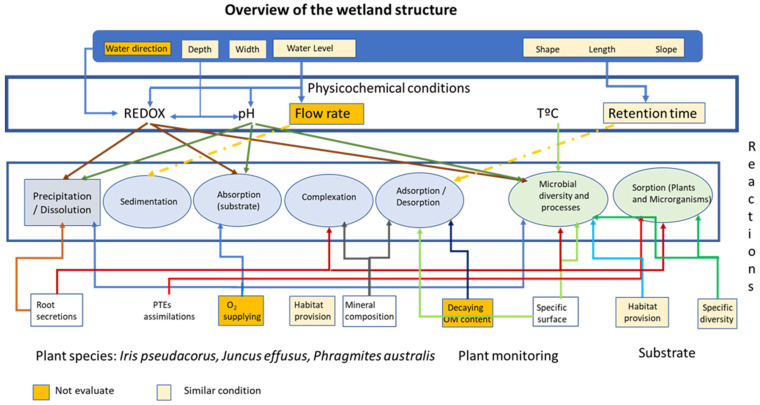
Graphic diagram of a wetland and main associated physicochemical processes.

Figure 2 summarises the key parameters to consider when designing a wetland [39], including the physicochemical conditions, the processes involved, and main interrelationships between them. Applying this scheme to the experiment described in Section 3, it can be seen that some parameters were not evaluated, such as water direction, flow rate, and O_2_, while others were kept constant, including depth and water level. Additionally, some parameters were controlled, such as substrate composition and PTE assimilation.

The experiment monitored the processes related to the substrate and evaluated the mobility of PTEs under different conditions and mineralogical compositions using various extracting media. The wetland’s behaviour in terms of PTE retention, whether through precipitation, absorption, complexation, or biological processes, was also monitored.

**Figure 3:**
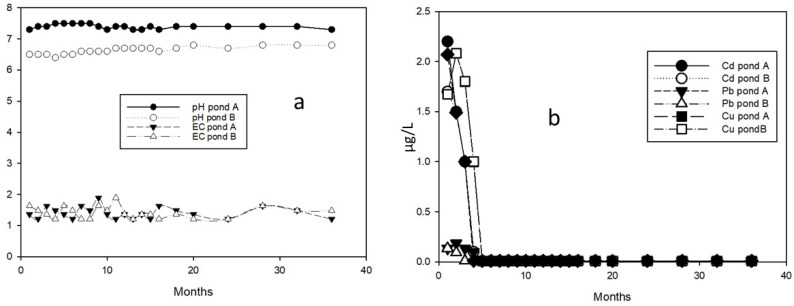
Monitoring of the waters of the two ponds A (the surface is covered with black sand) and B (the surface is covered with yellow sand and 30% limestone filler): (**a**) Monthly variation in electrical conductivity and pH. (**b**) Variation in the concentration of PTEs during the experiment.

The physicochemical and redox conditions, as well as the pH and temperature, were monitored throughout the two-year experiment, which included water sampling and monthly analysis. Figure 3a,b presents the results of the monitoring, indicating no significant variations during the study period.

The experiment used three plant species: *Iris pseudocorus*, *Juncus effusus*, and *Phragmites australis*. The monitoring plan was applied to the plants, and Table 5 shows the mean values of PTEs obtained for roots, aerial parts, and rhizosphere of the selected plants. These values provided data on PTE transfer to the plant, differentiating behaviours according to the type of plant and substrate [40,41]. Criteria for plant selection included tolerance to potentially toxic elements (PTEs) and a transfer factor of less than one to prevent the accumulation of PTEs in the shoots. This would prevent ingestion by animals and their incorporation into the food chain [42].

During the first year of the experiment, the plant species used (*Iris pseudacorus*, *Juncus effusus*, and *Phragmites australis*) showed a high degree of tolerance to the different mixtures tested, with good vegetative development. The plant species studied showed tolerance to Zn, Pb, and As, but their behaviour towards Cu was unclear. These species are suitable for wetlands and can be considered phytostabilising plants. They do not concentrate PTEs in their aerial parts, which means they can be safely incorporated into the food chain [43].

Regarding the root data obtained in the second year (see Table 5), the black and yellow sands have higher values compared to the controls, except for Fe, regardless of the plant species. As for the aerial part, the results varied significantly between metals and depended on the plant species.

The results can be attributed to the fact that the iron content in the root is conditioned by the formation of plaques or concretions, which is one of the defence mechanisms that these plants use to overcome anaerobic stress. This provokes an internal flow of oxygen through the plant and stabilises the iron plaques [44,45,46]. Generally, the highest metal root values were found in *Phragmites australis*, which had the highest values of Fe, followed by *Juncus effusus* and lastly *Iris pseudocorus*.

**Figure 4:**
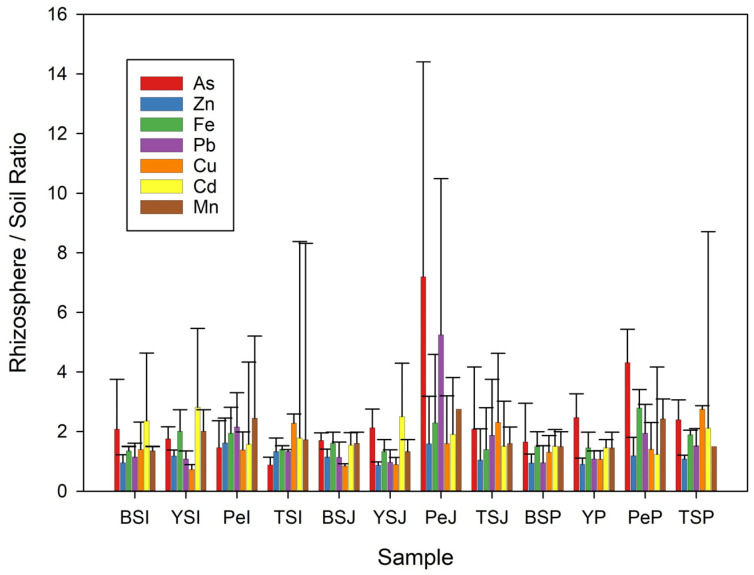
Average rhizosphere–soil ratio in BS (black sand), YS (yellow sand), Pe (peat), and TS (topsoil), respectively. *Iris pseudocorus*: samples BSI, YSI, PeI, and TSI. *Juncus effusus*: samples BSJ, YSJ, PeJ, and TSJ. *Phragmites australis*: samples BSP, YSP, PeP, and TSP.

The rhizosphere’s role as a concentrator of PTEs was evident from testing the rhizosphere–soil ratio for each sample. Figure 4 summarises the variations that occur in different plants and media. The treatments are classified into four groups: BS, which contains black sand; YS, which contains yellow sand with 30% limestone filler; Pe, which contains ≥50% peat; and TS, which contains more than 50% topsoil. The values exceeded one in nearly all cases, indicating a highly positive rhizosphere activity. The rhizosphere–soil ratio for both As and Pb was highest in the control treatments with their high peat content, with *juncus effusus* followed by *phragmites australis* having the highest values.

The principal component analysis grouped the PTE contents in the plant, rhizosphere, and soil into two rotated factors. One factor included Fe, As, Pb, Cu, Mn, and yellow sand content (D1), while the other factor included Zn, Cd, Cu, and black sand content (D2).

**Figure 5:**
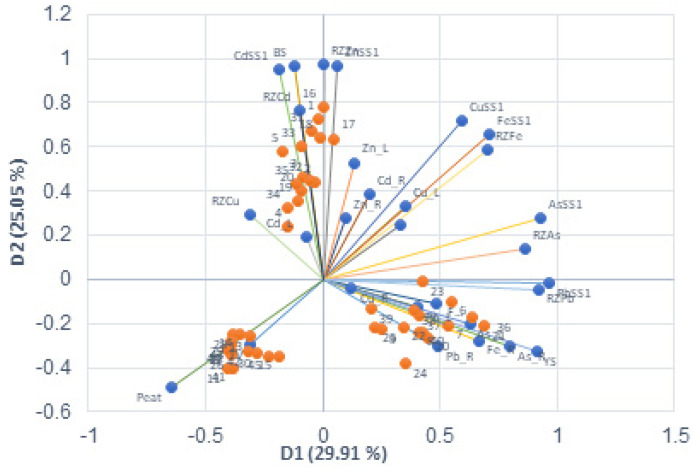
Biplot (D1 and D2 axes). Graphical plots of the variables and active observations. The variables (blue dots) are the following: PTESS1 (PTE soil content); RzPTE (PTE rizosphere content); PTE_R (PTE root content); PTE_L (PTE leaf content); BS (black sand); YS (yellow sand); Peat; and TS (topsoil). Samples (orange dots) are represented by numbers (1–45).

Peat and topsoil were found in the first factor with a negative sign. Figure 5 shows the factor scores of the samples and variables used. The experiment results display the separation of the three groups based on the presence of sand type and peat. Quadrant two represents samples with black sand (1–5, 16–20, and 31–35), quadrant three represents control samples (negative for peat and topsoil) (11–15, 26–30, and 41–45), and quadrant four represents yellow sands (positive for factor one and negative for peat) (6–10, 21–25, and 36–40).

Other principal component analyses, which consider a greater number of factors, may better explain the variance and separate more variables. However, they do not modify the essential finding that the samples are closely related with the type of substrate used. The characteristics of the initial materials are determinant as regards plant behaviour.

Wetland plants transfer oxygen from their stems to their roots and rhizomes through their internal gas spaces, known as aerenchyma. This process alters the redox conditions of the system, promoting oxidising reactions leading to the oxidation and precipitation of Fe^+3^ and Mn^+2^ [47]. Depending on the pH and Eh conditions of the wetland, Fe^+2^, Al, and Mn can form a wide variety of oxides and hydroxides [48,49]. The presence of organic complexing agents or microbial reactions can modify the equilibrium of Fe^+3^, which is normally immobilised at pH > 3, and affect its uptake.

Additionally, the precipitation of metals, such as carbonates, is a common mechanism for Ni, Pb, Mn, and Cu [50]. The immobilisation of metals such as Cu, Pb, Zn, Cd, and As through sulphide precipitation always requires anaerobic conditions and the presence of sulphate-reducing bacteria. This process forms sulphides, which are much less soluble than other chemical species [51,52,53].

**Figure 6:**
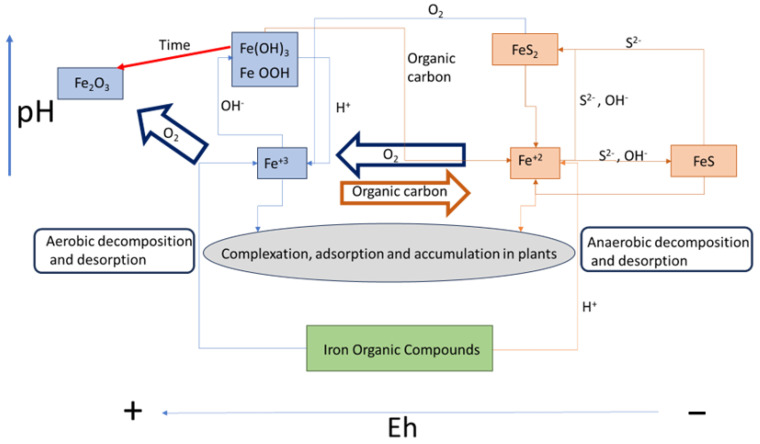
Behaviour of Fe in a wetland receiving mine drainage under different environmental conditions (pH and Eh) and the different processes that take place: oxidation, reduction, filtration, adsorption, plant assimilation, and biogeochemical precipitation.

The effectiveness of the wetland in question can be observed through two indicators: the vegetative growth of the plants and the development of biofilm during the first year of operation. All plant species cultivated in YS that participated in the experiment developed Fe plaques to varying degrees as a defence against oxidative stress. No plaque development was observed in pots with BS mixtures, possibly due to the media being more porous and less saline [54,55]. This process was observed in the three selected species, which collaborated in the immobilisation of PTEs in the rhizosphere, retaining significant amounts of Pb, Si, and As [56]. Figure 6 summarises the main reactions and mechanisms that occurred in the wetland in response to the results obtained in the experiment.

The black sands favoured aerobic conditions, while the yellow sands had a finer texture and lead to anaerobiosis, resulting in higher mobility of Fe and Mn. The presence of limestone filler neutralises the pH, causing the precipitation of Fe(III) and increasing the mobilised As uptake. These two processes may conflict with each other, and the microbial population may shift the balance to one side or the other. Organic matter is an important factor in promoting anaerobiosis and acting as a complexing agent.

**Figure 7:**
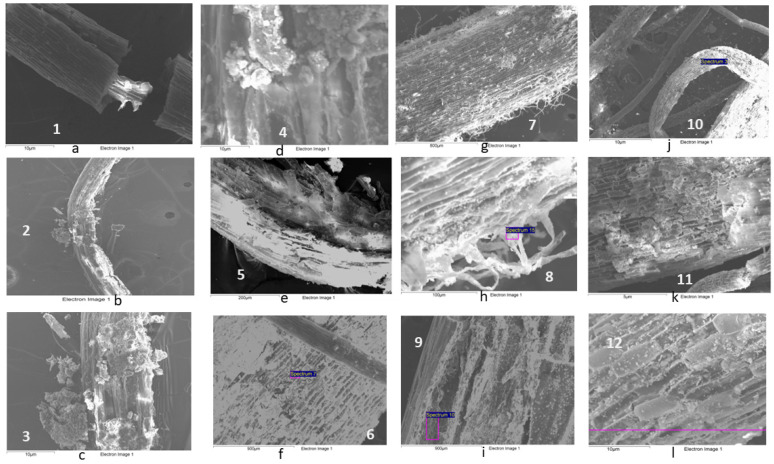
SEM pictures from *Iris pseudacorus*: (**a**) Split root (100×). (**b**) Root (55×). (**c**) Plate area in b (250×). (**d**) Iron plate (1500×). *Juncus effusus*: (**e**) Root (220×). (**f**) Magnification of e (1500×). (**g**) Root (100×). (**h**) Magnification of g (500×). *Phragmites australis*: (**i**) Plate zone (500×). (**j**) Root (250×). (**k**) Magnification of 10 (500×). (**l**) Root surface plate (1000×).

One of the most characteristic processes in wetlands is the precipitation of iron hydroxide, either in the substrate or as part of plaques in plant roots. In this study, we identified plaques formed in the roots of all types of plants used. The composition was mostly Fe oxide, but sometimes included significant amounts of Si and S, among others. Table 6 shows that the most frequent PTEs associated with the plaques were As, Cu, and Pb. Plaques tended to form in the roots of the three selected plants, particularly in those growing in yellow sands where surface oxidation crusts can develop due to water level variations. Figure 7 displays examples of plaque in roots observed by SEM-EDX, as well as spot analysis. The observed morphology may depend on the particular plant, and it is more frequently found in thin roots or adapted to the root surface, as shown in Figure 7f,l for *Juncus effusus* and *Phragmites australis*, respectively.

## 3. Materials and Methods

### 3.1. Selection of Materials Used in the Experiment

Description of materials

- Limestone filler (LF)

LF is a residue of limestone aggregates used in construction, with a granulometry lower than 63 µm, which poses an environmental problem in the quarries where it is produced. Its mineralogical and chemical characteristics have proved appropriate for the use as a pH stabiliser and PTE immobiliser [57].

- Black Sand (BS)

BS is a granoclassified sediment deposited by the sea and reaches the beach by the mineral washing muds. It has a sandy texture and a very stable and not very reactive mineralogical composition, as shown by studies of forced alterability under climatic conditions such as the present ones by applying a 300-year model [57].

- Yellow Sand (YS)

This sediment is dumped directly on the surface of the bay, with a fine silty-clayey texture, variable pH, and it is sometimes very acidic, especially when it is in the first 50 cm. It undergoes supergene alteration. Its mineralogical composition corresponds to poorly crystallised jarosites, soluble salts, Fe hydroxides, sulphides, etc. This sediment presents different states of alteration and it is not as homogeneous a material as black sands.

- Topsoil (TS)

The soil used in the experiment was selected from areas close to the site that were minimally impacted by mining. A cultivated soil with low electrical conductivity values and an organic matter content higher than 1.5% [35], without significant values of PTEs, was chosen.

- Peat

The peat used in mixtures is a commercial gardening product consisting of black peat and vegetal compost in a 1:1 ratio. It has an organic carbon content of 55%, a moisture content of 45%, a pH of 7.2, and a C/N ratio of 25. Additionally, it has non-significant levels of PTEs [58].

### 3.2. Construction of Ponds

As suggested by Wildeman et al. [59], different stages in wetland design should be considered: (1) laboratory studies, (2) prototypes, (3) pilot-scale systems, and (4) large-scale systems. Each of the stages allows for the provision of important and valuable information for the development of the optimal systems to be executed on a real scale.

**Figure 8:**
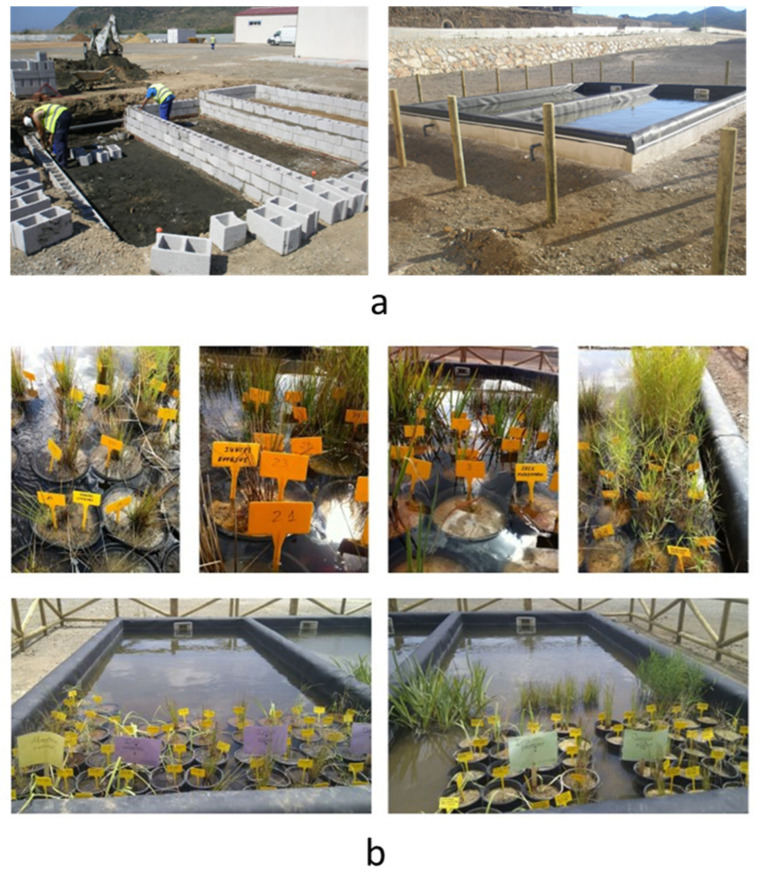
(**a**) Construction phases of the experimental wetland. (**b**) Panoramic view of the ponds and detail of the pots used.

The design of the ponds was an adaptation of the scheme corresponding to Figure 1, but with two ponds (A and B), both measuring 7 × 3 m. The bottom was water-proofed with a PVC coating to prevent soil contamination. In the constructed wetlands, the depth of the filter beds is normally 60–80 cm with a bottom slope of 0.5–1% from the inlet to the outlet to allow for good drainage [60], thus achieving a hydraulic retention time and a specific surface area of 2–5 days and approximately 2–5 m^2^/PE (m^2^/person equivalent), values which are sufficient for discharge into water [60,61]. In order to simulate different possibilities in the final wetland, each possibility was tested at three different depths: 20 cm, 40 cm, and 80 cm. Figure 8 shows two stages of the pond construction. Pond A was filled with a single material (BS) at its deepest point (80 cm), while pond B was filled with two layers: one of BS and one of YS stabilised with 30% LF (refer to Figure 8).

### 3.3. Development of the Experiment

The experiment was developed in two phases: a first phase with materials without mixtures, using the two types of sands and plant soil as a control with a total of 9 pots [43], and a second phase with different variations with a total of 45 pots [42]. In both phases, all experiments were carried out in triplicate, except for the controls of plant soil that were tested in six replicates.

The containers were positioned in the ponds with the water level at the container edge, replicating wetland conditions. All pots were placed on the deepest step (80 cm), in repetitions of 3 specimens for each of the species (see Figure 8). In all cases, the pots were filled with substrates made with different mixtures of plant soil and/or peat, with black or yellow sands, according to the mixing percentages indicated in Table 7. The 30% limestone filler was added to the yellow sand used.

### 3.4. Experiment Monitoring Plan

The ponds were filled with water from the local supply. Weekly monitoring of the water’s PTEs content, pH, and EC was conducted, followed by monthly sampling until the end of the experiments.

The study monitored the vegetative development, rhizosphere, root, and aerial parts of the plant. Vegetative development was monitored by quarterly measurements. Total PTEs were measured annually in the rhizosphere, roots, and aerial parts of each plant species in each pot.

### 3.5. Analytical Methodology

Soil samples (rhizosphere) were air-dried and sieved through a 2 mm screen for general analytical determinations. The pH and EC were determined in a 1:5 (m/V) suspension of soil in deionised water (resistivity ≥ 18 MΩ cm). To determine the total PTE content, the soil samples were first ground to a fine powder using a zirconium ball mill. Aliquots (0.1 g) of soil samples were placed in Teflon vessels, and a mixture of 5 mL concentrated HF (37%), 200 μL concentrated HNO_3_ (65%), and 5 mL water was added. When digestion in the microwave system was complete, the samples were transferred to a volumetric flask and brought to 50 mL before measurement. Teflon or other suitable plastic ware was used for handling these liquids.

Fresh plant materials were separated into the root and above-ground part, carefully washed with fresh water, cleaned using an ultrasonic bath to remove dust contamination, and finally rinsed with deionised water, air-dried, and then lyophilised. Then, 200 mg of lyophilised vegetal tissue was placed in Teflon vessels with 3 mL water, 2 mL concentrated H_2_O_2_, and 5 mL concentrated HNO_3_ acid solution and subjected to digestion in a microwave oven, finally obtaining 50 mL solutions, which were analysed.

The reliability of the results was assessed through analysis of the NIST standard reference material (SRM 2711 Montana Soil). Spikes, duplicates, and reagent blanks were also used as a part of the quality control.

Zn, Mn, and Fe content in soils were determined by flame atomic absorption spectrometry (FAAS). Pb, Cd, and Cu contents were determined by electrothermal atomisation atomic absorption spectrometry (ETAAS) using a high-resolution continuum source ContrAA spectrometer from Analytik Jena AG. Arsenic was measured by atomic fluorescence spectrometry using an automated continuous flow hydride generation system (PSA Millenium Merlin 10055). The limit of quantification for the selected elements was 0.3 μg kg^−1^ for As, 10 μg kg^−1^ for Pb, 0.5 μg kg^−1^ for Cu, 0.6 μg kg^−1^ for Cd, 50 μg kg^−1^ for Zn and Mn, and 100 μg kg^−1^ for Fe.

A mobilisation study was conducted using a simple extraction method to estimate the behaviour of sediments under different environmental conditions and the natural or potential mobility of PTEs. Extractant reagents were used to simulate various environmental conditions, including exposure to rain and contact with an acidic pH medium (AMD). The experiment also simulated more extreme conditions with changes in redox potential, and the extracted PTEs were then determined for each condition. Four chemical reagents were used for extraction, namely, water medium (1:5 extract), nitric acid medium (1 g of solid in 50 mL 0.1 M HNO_3_), complexing–reducing medium, Merha–Jackson extraction [62] using sodium citrate (complexing agent) and dithionite (reducing agent), and an oxidising medium (sequential extraction procedure, step 3, BCR (Community Bureau of Reference)) [63]. The procedure begins by adding 40 mL of H_2_O_2_ (pH 2–3) to 1 g of the sample. The mixture is then kept at room temperature for an hour. Afterward, digestion is continued in a bath at 85 °C until the volume is reduced to a few millilitres. The above procedure is repeated adding 10 mL of H_2_O_2_ (pH 2–3). After that, 50 mL of 1 M NH_4_OAc (pH 2) is added and the mixture is stirred for 16 h at 22 ± 5 °C. Finally, the extract is separated from the residue by centrifugation at 3000 rpm for 20 min.

After filling the ponds, the PTE content and the pH and EC of the water were determined. The water samples were filtered at 45 µm and kept at 4 °C for later analysis in the laboratory. The PTE determinations were carried out using the methodology described for the solid samples.

A semiquantitative estimation of the mineralogical composition of the solid samples was made by powder X-ray diffraction (XRD) analysis, using Cu-Kα radiation with a PW3040 Philips Diffractometer.from Philips Corporation, Netherlands X-powder software, ver. 2021.04.24, as used to analyse diffractograms [64]. The powder diffraction file (PDF2) database was used for peak identification.

A detailed study of the fate of each PTE in the plant was conducted using scanning electron microscopy (SEM) [35,36,37,38,39,40,41,42,43]. As a complementary technique, the vegetal samples were also studied by microscopy (SEM-EDX). The samples were first freeze-dried and then coated with carbon. The analysis was carried out with a JEOL-6100 scanning microscope nd an Oxford Instrument INCA X-ray dispersion Microanalysis System provided with an X-ray and backscattered electron detector and a digital image capture system from JEOL (EUROPE) SAS, Paris, France.

### 3.6. Statistical Analysis

The statistical study was carried out with the XSTAT 2023.2.1414 program. Descriptive statistics (mean, percentiles, and standard deviation, among others) of the results obtained and a principal component analysis with varimax rotation were carried out to group the different variables and define the behaviour of the samples according to them.

## 4. Conclusions

The construction of a wetland as a pilot experiment to stabilise soils near the sea and affected by the course of a rambla can be considered a viable option for the recovery of soils affected by mining.

It is possible to use materials from the site with or without recovery measures, but a study of the materials to be used is needed beforehand to evaluate their future behaviour in scenarios other than the current ones. This study used the two most representative types of sediment in the area studied: yellow sand and black sand. Yellow sand, which has a fine texture and acid pH, was stabilised with limestone filler and mixed with plant soil and organic matter to encourage plant growth. Black sand was used without any limestone amendments but was mixed with peat and plant soil.

These experiments enable a comparison of the behaviour of three common plant species in wetlands under different scenarios and substrates. The presence of organic matter, such as peat, favours the retention of metals, particularly As and Pb, and plant growth. However, it can pose a problem during drought conditions, as the substrate disappears and the PTEs are released, becoming mobile under oxidising conditions that have the potential to mobilise them. The experiment enabled the modelling of the future material behaviour in the area as a support for a wetland. This is achieved once the surrounding soils have been reclaimed and vegetation is capable of thriving under extreme conditions. The vegetation buffers the impact of AMD in the marine environment and retains PTEs without translocating them to the aerial parts, thus reducing the risk of ingestion in the trophic chain.

## Figures and Tables

**Table 1:** Physicochemical characteristics of the selected limestone filler (LF): average and standard deviation.

pH	Eh (mV)	Electric Conductivity (mS·cm^−1^)	Mean Particle Size (µm)	Textural Class USDAas	BET (m^2^·g^−1^)	Permeability (cm·h^−1^)	Relative Density g·cm^−3^	Porosity (%)
9.02 ± 0.09	150 ± 0.4	0.22 ± 0.04	55 ± 1	silt	0.70 ± 0.3	1.04 ± 0.32	2.30 ± 0.05	17.0 ± 0.1

Eh (mV): electrochemical potential.

**Table 2:** Physicochemical characteristics of selected materials: average and standard deviation.

Sample	pH	EC (dS/m)	OM%	Clay%	Silt%	Sand%
BS	7.46 ± 0.08	2.44 ± 0.22	0.17 ± 0.08	-	2.00 ± 0.55	98.00 ± 0.60
YS	5.05 ± 0.10	18.43 ± 3.57	<QL	0.19 ± 0.09	10.41 ± 2.57	81.20 ± 3.05
TS	8.10 ± 0.25	2.0 ± 0.32	1.8 ± 0.21	7.51 ± 1.35	40.59 ± 3.02	52.00 ± 2.83
LF	9.02 ± 0.09	0.22 ± 0.04	<QL	20.88 ± 4.43	71.2 ± 3.51	8.00 ± 2.70

EC: electric conductivity; OM: organic matter content.

**Table 3:** Average and standard deviation of mineralogical composition (%).

Sample	Phyl. 14 Å	Phyl. 10 Å	Quartz	Albite	Calcite	Dolomite	Siderite	Akageneite	Hematite	Gypsum	Jarosite	Pyrite	Magnetite	Goethite	Greenalite	Copiapite
BS	14 ± 2	6 ± 2	7 ± 2				27 ± 3	12 ± 3	5 ± 3	2 ± 2		9 ± 3	6 ± 3	6 ± 2	7 ± 2	
YS	8 ± 1	12 ± 2	8 ± 4					10 ± 2	5 ± 2	8 ± 2	36 ± 6		5 ± 3	6 ± 2	6 ± 2	16 ± 10
TS	4 ± 2	30 ± 6	12 ± 2	2 ± 2	34 ± 2	18 ± 5			2 ± 2	5 ± 2						
LF		3 ± 1	4 ± 2		60 ± 5	33 ± 5										

**Table 4:** Average and standard deviation of potential toxic elements’ content in the mixtures used in the experiment.

Sample	As (mg/kg)	Pb (mg/kg)	Cd (mg/kg)	Cu (mg/kg)	Fe (%)	Zn (mg/kg)	Mn (mg/kg)
1 *	202 ± 20	670 ± 25	8 ± 1	31 ± 1	25.90 ± 0.5	8993 ± 87	7360 ± 89
2	155 ± 17	516 ± 19	6 ± 1	26 ± 1	20.47 ± 0.7	7032 ± 56	2500 ± 110
3	152 ± 19	504 ± 22	6 ± 1	28 ± 1	19.68 ± 0.8	6855 ± 68	2432 ± 121
4	109 ± 12	361 ± 18	5 ± 1	22 ± 2	15.00 ± 0.9	5072 ± 49	1852 ± 45
5	102 ± 14	337 ± 20	5 ± 1	25 ± 2	13.42 ± 0.8	4717 ± 59	4525 ± 225
6 **	333 ± 35	2037 ± 59	1 ± 0.3	28 ± 1	24.07 ± 1.0	2396 ± 65	320 ± 54
7	253 ± 25	1541 ± 45	1 ± 0.2	24 ± 2	19.07 ± 0.7	2085 ± 43	423 ± 49
8	250 ± 28	1529 ± 54	1 ± 0.4	25 ± 1	18.28 ± 0.5	1907 ± 56	529 ± 31
9	174 ± 16	1045 ± 32	1 ± 0.3	20 ± 1	14.06 ± 0.6	1773 ± 48	520 ± 46
10	167 ± 19	1021 ± 25	1 ± 0.3	23 ± 2	12.48 ± 0.7	1418 ± 36	360 ± 28
11 ***	1 ± 0.6	4 ± 2	1 ± 0.1	18 ± 1	0.90 ± 0.2	440 ± 20	105 ± 17
12	5 ± 2	16 ± 3	1 ± 0.1	17 ± 1	1.69 ± 0.4	618 ± 32	250 ± 34
13	8 ± 2	28 ± 3	1 ± 0.2	15 ± 1	2.48 ± 0.5	795 ± 30	450 ± 28
14	12 ± 2	40 ± 2	1 ± 0.2	14 ± 1	3.27 ± 0.6	973 ± 48	747 ± 59
15 ****	15 ± 3	52 ± 4	1 ± 0.2	12 ± 1	4.06 ± 0.7	1150 ± 78	840 ± 52

1 *: 100% BS; 6 **: 100% (YS + 30% LF); 11 ***: 100% Peat; 15 ****: 100% TS.

**Table 5:** Average contents and standard deviation of rhizosphere, root and leaf.

	*Iris* *pseudocorus*		*Juncus effusus*		*Phragmites australis*		*Iris* *pseudocorus*		*Juncus effusus*		*Phragmites australis*		*Iris* *pseudocorus*		*Juncus effusus*		*Phragmites* *australis*	
	Black Sand						Yellow Sand						Control					
Root																		
	Media	SD	Media	SD	Media	SD	Media	SD	Media	SD	Media	SD	Media	SD	Media	SD	Media	SD
Cu_R	22	8	23	3	19	3	16	15	29	15	33	25	20	18	20	7	21	11
Fe_R	0.44	0.28	0.54	0.50	0.46	0 42	0.29	0.17	4.12	2.68	3.16	2.94	0.82	0.12	0.57	0.50	0.58	0.12
Pb_R	33	27	59	54	16	9	169	94	248	140	125	96	18	18	21	11	23	22
As_R	37	32	11	6	14	12	272	164	225	212	409	145	15	14	21	17	16	20
Zn_R	624	305	723	645	514	385	284	130	291	183	521	234	176	113	373	332	132	59
Cd_R	2	2	1	1	1	1	<Q.L		1		1	1	1		1		<Q.L	
Mn R	228	214	296	206	566	520	85	109	246	189	471	397	331	326	216	130	172	164
Leaf																		
Cu_L	8	4	17	4	13	3	11	5	11	6	12	7	10	9	7	2	8	3
Fe_L	356	145	543	377	461	357	2231	3346	1105	822	634	270	368	153	160	73	188	63
Pb_L	7	7	8	5	4	4	14	10	17	11	4	3	6	4	2	1	4	5
As_L	4	7	2	3	1	0.5	13	13	10	9	4	5	1	0.5	1	0.5	1	1
Zn_L	132	57	333	143	248	100	131	105	156	112	152	43	143	80	138	41	106	18
Cd_L	<Q.L		1	0.1	<Q.L		<Q.L		<Q.L		<Q.L		<Q.L		<Q.L		<Q.L	
Mn L	43	36	163	92	169	37	87	27	74	52	126	65	205	73	100	94	138	87
Rhizosphere																		
As_Rz	202	54	234	50	220	125	374	72	268	120	527	99	7	6	26	12	24	11
Zn_Rz	6080	1235	7031	827	6054	1013	1500	150	1526	168	1633	322	116	47	101	13	93	39
Fe_Rz	26.88	5.05	28.83	4.27	27.36	4.73	20.05	4.07	21.66	4.66	23.78	5.77	4.21	1.85	4.34	0.18	5.42	1.84
Pb_Rz	482	206	483	170	449	209	1473	179	1261	235	1473	179	47	25	66	21	48	27
Cu_Rz	26	4	22	3	30	14	17	4	20	8	26	3	26	7	27	4	27	11
Cd_Rz	9	2	9	1	9	2	2	2	1	0.4	1	0.4	2	1	2	0.5	2	1
Mn Rz	4840	3300	3659	2246	7048	2921	436	163	437	120	418	167	633	293	675	280	644	325

Root (R), leaf (L) and rhizosphere (Rz). Fe_R and Fe_Rz are expressed in %. SD = standard deviation.

**Table 6:** Microanalysis results in roots (Weight %).

Element	c *Iris pseudacorus*	d *Iris pseudacorus*	f *Juncus effusus*	h *Juncus effusus*	i *Phragmites australis*	l *Phragmites australis*
O	17.27	27.36	28.06	29.68	11.38	32.74
Na	2.70	3.16	4.01	4.66	0.96	2.11
Mg	1.34	0.71	1.43	1.82	2.41	2.78
Al	0.68	0.71	1.26	1.66	3.13	9.55
Si	7.93	8.61	3.57	3.95	11.71	17.14
P	0.46	0.20	0.27	0.20	0.22	0.28
S	1.75	1.98	13.35	14.26	6.73	2.45
Cl	5.49	6.46	1.76	1.51	3.32	1.65
K	4.59	5.31	3.80	5.71	3.71	4.69
Ca	2.46	1.99	1.53	2.13	7.48	2.73
Mn	0.52	0.20	0.12	0.16	0.07	0.23
Fe	53.50	40.29	36.85	30.21	44.02	23.14
Cu	0.40	1.12	0.27	0.34	0.32	0.26
Zn	0.50	0.40	0.28	0.40	0.73	0.30
As L	2.43	0.66	0.59	0.71	0.57	0.66
Pb	0.22	2.82	2.87	2.64	2.28	0.20
Cd	0.30	0.15	0.50	0.13	0.55	32.74

**Table 7:** Mixtures used in the experiment.

	BS (%)	YS (%)	TS (%)	Peat (%)	LF
1	100				
2	75		25		
3	75			25	
4	50		50		
5	50			50	
6		70			30
7		52.5	25		22.3
8		52.5		25	22.3
9		35	50		15
10		35		50	15
11				100	
12			25	75	
13			50	50	
14			75	25	
15			100		

BS: black sand; YS: yellow sand; TS: topsoil; LF: limestone filler.

## Data Availability

The data presented in this study are available on request from the corresponding author.
